# 4-Nitro­phenol–2,4,6-triamino-1,3,5-triazine–water (2/1/1)

**DOI:** 10.1107/S1600536812029066

**Published:** 2012-06-30

**Authors:** N. Kanagathara, G. Chakkaravarthi, M. K. Marchewka, S. Gunasekaran, G. Anbalagan

**Affiliations:** a Department of Physics, Vel Tech Multi Tech Dr. Rangarajan Dr. Sakunthala Enginering College, Avadi, Chennai 600 062, India; b Department of Physics, CPCL Polytechnic College, Chennai 600 068, India; c Institute of Low Temperature and Structure Research, Polish Academy of Sciences, 50-950 Wrocław, 2, PO Box 937, Poland; d PG and Research Department of Physics, Pachiayappa’s College, Chennai 600 030, India; e Department of Physics, Presidency College, Chennai 600 005, India

## Abstract

In the title adduct, 2C_6_H_5_NO_3_·C_3_H_6_N_6_·H_2_O, the melamine and the two independent nitrophenol molecules are essentially planar, with maximum deviations of 0.0294 (10), 0.0706 (12) and 0.0742 (12) Å, respectively. In the crystal, N—H⋯N, O—H⋯N, N—H⋯O and O—H⋯O hydrogen bonds link the components into a three-dimensional network. In addition, weak π–π inter­actions [centroid–centroid distances = 3.728 (3) and 3.749 (3) Å] are observed.

## Related literature

For applications of melamine, see: Cook *et al.* (2005 ▶); Rima *et al.* (2008 ▶). For a related structure, see: Cousson *et al.* (2005 ▶).
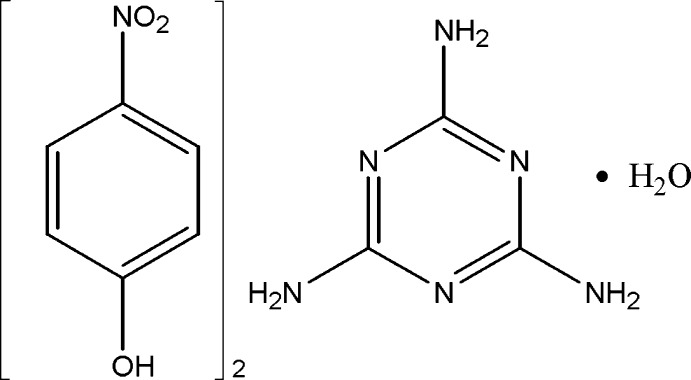


## Experimental

### 

#### Crystal data

2C_6_H_5_NO_3_·C_3_H_6_N_6_·H_2_O
*M* *_r_* = 422.37
Triclinic, 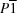

*a* = 7.123 (5) Å
*b* = 10.577 (4) Å
*c* = 13.680 (5) Å
α = 68.256 (5)°
β = 88.772 (6)°
γ = 76.604 (5)°
*V* = 928.9 (8) Å^3^
*Z* = 2
Mo *K*α radiation
μ = 0.12 mm^−1^
*T* = 295 K
0.30 × 0.20 × 0.20 mm

#### Data collection

Bruker Kappa APEXII diffractometer
Absorption correction: multi-scan (*SADABS*; Sheldrick, 1996 ▶) *T*_min_ = 0.964, *T*_max_ = 0.976
21610 measured reflections
5696 independent reflections
4164 reflections with *I* > 2σ(*I*)
*R*_int_ = 0.030

#### Refinement

*R*[*F* ^2^ > 2σ(*F* ^2^)] = 0.043
*wR*(*F* ^2^) = 0.122
*S* = 1.03
5696 reflections
311 parameters
H atoms treated by a mixture of independent and constrained refinement
Δρ_max_ = 0.20 e Å^−3^
Δρ_min_ = −0.25 e Å^−3^

### 

Data collection: *APEX2* (Bruker, 2004 ▶); cell refinement: *SAINT* (Bruker, 2004 ▶); data reduction: *SAINT*; program(s) used to solve structure: *SHELXS97* (Sheldrick, 2008 ▶); program(s) used to refine structure: *SHELXL97* (Sheldrick, 2008 ▶); molecular graphics: *PLATON* (Spek, 2009 ▶); software used to prepare material for publication: *SHELXL97*.

## Supplementary Material

Crystal structure: contains datablock(s) global, I. DOI: 10.1107/S1600536812029066/lh5493sup1.cif

Structure factors: contains datablock(s) I. DOI: 10.1107/S1600536812029066/lh5493Isup2.hkl

Supplementary material file. DOI: 10.1107/S1600536812029066/lh5493Isup3.cml

Additional supplementary materials:  crystallographic information; 3D view; checkCIF report

## Figures and Tables

**Table 1:** Hydrogen-bond geometry (Å, °)

*D*—H⋯*A*	*D*—H	H⋯*A*	*D*⋯*A*	*D*—H⋯*A*
O1—H1⋯O7^i^	0.90 (2)	1.76 (2)	2.6600 (18)	172 (2)
O4—H4⋯N5	0.91 (2)	1.87 (2)	2.7217 (16)	157 (2)
O7—H7*A*⋯N4	0.88 (2)	1.94 (2)	2.8020 (18)	166 (2)
O7—H7*B*⋯O2^iv^	0.84 (2)	2.22 (2)	3.0424 (18)	164 (2)
N6—H6*A*⋯O6^ii^	0.860 (18)	2.363 (19)	3.0276 (16)	134 (2)
N6—H6*B*⋯N3^iii^	0.845 (18)	2.235 (19)	3.080 (2)	178 (2)
N7—H7*C*⋯O6^v^	0.867 (17)	2.250 (17)	3.056 (2)	155 (2)
N7—H7*D*⋯O1^v^	0.894 (19)	2.049 (19)	2.8996 (17)	159 (2)
N8—H8*A*⋯O3	0.830 (17)	2.367 (18)	3.158 (2)	159 (2)
N8—H8*B*⋯O7^iv^	0.867 (19)	2.517 (18)	3.1890 (19)	135 (2)

Symmetry codes: (i) 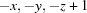; (ii) 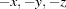; (iii) 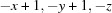; (iv) 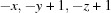; (v) 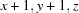.

**Fig. 1.**
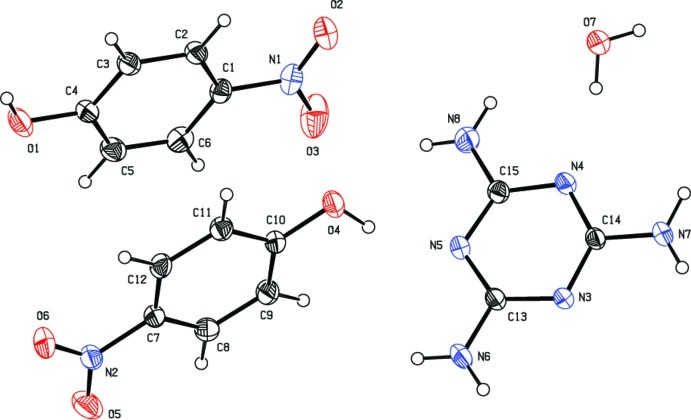
The molecular structure of (I), with 30% probability displacement ellipsoids for non-H atoms.

**Fig. 2.**
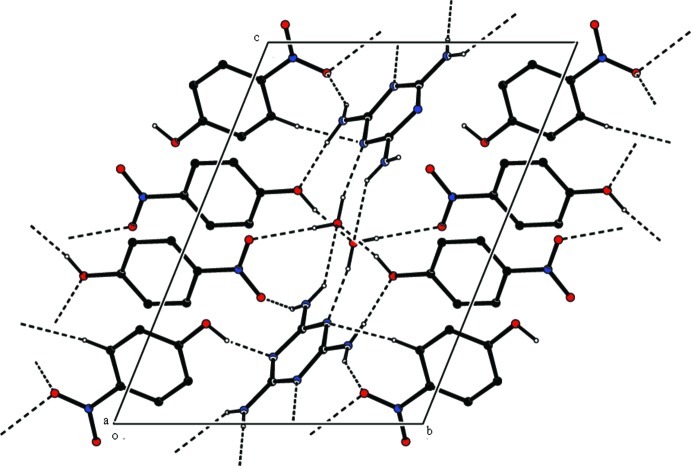
The packing of (I), viewed along the a axis. Intermolecular Hydrogen bonds are shown as dashed lines. H atoms not involved in hydrogen bonding have been omitted.

## supplementary crystallographic information

### Comment

Melamines are used in the production of melamine foam in polymeric cleaning (Rima *et al.*, 2008) and as a chemical intermediate in plastics manufacturing (Cook *et al.*, 2005). Here, we report the crystal structure of a the title compound. The asymmetric unit contains one melamine molecule, two independent nitrophenol molecules and one solvent water molecule.

The geometric parameters of the melamine molecule (I) (Fig. 1) are comparable with those determined by Cousson *et al.* (2005). The melamine and nitrophenol molecules are essentially planar, with a maximum deviation of -0.0294 (10) Å for atom N4 in the least square plane (N6/C13/C14/N7/N4/C15/N8/N5), -0.0706 (12) Å for atom O2 in the least square plane (O1/C1-C6/N1/O2/O3) and 0.0742 (12) Å for atom O5 in the least square plane (O4/C10/C11/C12/C7/C8/C9/N2/O5/O6).

In the crystal, O—H···N, N—H···O and O—H···O hydrogen bonds (Table 1 & Fig. 2) and π–π interactions [Cg1···Cg1 (1-x,-y,1-z) distance of 3.749 (3)Å; Cg1···Cg2 (x,-1+y,z) distance of 3.728 (3)Å and Cg2···Cg1 (x,1+y,z) distance of 3.728 (3)Å; Cg1 and Cg2 are the centroids of the rings (C1-C6) and (C7-C12), respectively] connect the components of the structure into a three-dimensional network.

### Experimental

Melamine (1.2612g, 0.01 mmol) was dissolved in 200 ml of hot solution of distilled water. p-Nitrophenol (1.3911g, 0.01 mmol) was dissolved in 100 ml of distilled water separately. To the hot solution of melamine, p-nitrophenol solution was added gently, and stirred well for nearly five hours to get the homogenous solution and the mixture is allowed to evaporate. Within a few days tiny, transparent, yellowish crystals were formed. Recrystallization was carried out by using distilled water to get the pure crystal suitable for X-ray diffraction.

### Refinement

The H atoms for aromatic C-H groups were positioned geometrically with C–H = 0.93 %A and allowed to ride on their parent atoms, with U_iso_(H) = 1.2U_eq_(C) and all other H atoms were located in a difference Fourier map and allowed to refine freely [N—H = 0.830 (17)-0.894 (19)Å and O—H = 0.84 (2)–0.91 (2)Å].

### Crystal data

**Table:**

2C_6_H_5_NO_3_·C_3_H_6_N_6_·H_2_O	*Z* = 2
*M**_r_* = 422.37	*F*(000) = 440
Triclinic, *P*1	*D*_x_ = 1.510 Mg m^−^^3^
Hall symbol: -P 1	Mo *K*α radiation, λ = 0.71073 Å
*a* = 7.123 (5) Å	Cell parameters from 21610 reflections
*b* = 10.577 (4) Å	θ = 2.1–30.7°
*c* = 13.680 (5) Å	µ = 0.12 mm^−^^1^
α = 68.256 (5)°	*T* = 295 K
β = 88.772 (6)°	Block, yellow
γ = 76.604 (5)°	0.30 × 0.20 × 0.20 mm
*V* = 928.9 (8) Å^3^	

### Data collection

**Table:**

Bruker Kappa APEXII diffractometer	5696 independent reflections
Radiation source: fine-focus sealed tube	4164 reflections with *I* > 2σ(*I*)
Graphite monochromator	*R*_int_ = 0.030
ω and φ scans	θ_max_ = 30.7°, θ_min_ = 2.1°
Absorption correction: multi-scan (*SADABS*; Sheldrick, 1996)	*h* = −8→10
*T*_min_ = 0.964, *T*_max_ = 0.976	*k* = −15→15
21610 measured reflections	*l* = −17→19

### Refinement

**Table:**

Refinement on *F*^2^	Primary atom site location: structure-invariant direct methods
Least-squares matrix: full	Secondary atom site location: difference Fourier map
*R*[*F*^2^ > 2σ(*F*^2^)] = 0.043	Hydrogen site location: inferred from neighbouring sites
*wR*(*F*^2^) = 0.122	H atoms treated by a mixture of independent and constrained refinement
*S* = 1.03	*w* = 1/[σ^2^(*F*_o_^2^) + (0.0598*P*)^2^ + 0.1276*P*] where *P* = (*F*_o_^2^ + 2*F*_c_^2^)/3
5696 reflections	(Δ/σ)_max_ < 0.001
311 parameters	Δρ_max_ = 0.20 e Å^−^^3^
0 restraints	Δρ_min_ = −0.25 e Å^−^^3^

### Special details

**Table:**

Geometry. All esds (except the esd in the dihedral angle between two l.s. planes) are estimated using the full covariance matrix. The cell esds are taken into account individually in the estimation of esds in distances, angles and torsion angles; correlations between esds in cell parameters are only used when they are defined by crystal symmetry. An approximate (isotropic) treatment of cell esds is used for estimating esds involving l.s. planes.

### Fractional atomic coordinates and isotropic or equivalent isotropic displacement parameters (Å^2^)

**Table:**

	*x*	*y*	*z*	*U*_iso_*/*U*_eq_	
C1	−0.33225 (16)	0.07164 (12)	0.40065 (10)	0.0341 (2)	
C2	−0.24544 (16)	−0.05332 (12)	0.48003 (9)	0.0348 (2)	
H2	−0.1702	−0.0549	0.5354	0.042*	
C3	−0.27246 (18)	−0.17603 (13)	0.47577 (9)	0.0370 (3)	
H3	−0.2136	−0.2617	0.5281	0.044*	
C4	−0.38715 (18)	−0.17217 (13)	0.39367 (10)	0.0378 (3)	
C5	−0.47281 (18)	−0.04548 (15)	0.31426 (10)	0.0423 (3)	
H5	−0.5486	−0.0437	0.2590	0.051*	
C6	−0.44573 (17)	0.07743 (14)	0.31718 (10)	0.0406 (3)	
H6	−0.5024	0.1630	0.2642	0.049*	
C7	−0.16167 (17)	−0.02771 (12)	0.09893 (9)	0.0345 (2)	
C8	−0.20456 (18)	0.11701 (13)	0.05818 (10)	0.0394 (3)	
H8	−0.2801	0.1679	−0.0049	0.047*	
C9	−0.13380 (19)	0.18491 (12)	0.11231 (10)	0.0387 (3)	
H9	−0.1618	0.2824	0.0860	0.046*	
C10	−0.02072 (16)	0.10786 (12)	0.20608 (9)	0.0331 (2)	
C11	0.01838 (17)	−0.03791 (12)	0.24690 (10)	0.0357 (2)	
H11	0.0921	−0.0892	0.3105	0.043*	
C12	−0.05206 (17)	−0.10608 (12)	0.19311 (10)	0.0361 (3)	
H12	−0.0263	−0.2035	0.2197	0.043*	
C13	0.26472 (17)	0.45692 (12)	0.11080 (9)	0.0339 (2)	
C14	0.36186 (16)	0.58004 (11)	0.19500 (9)	0.0305 (2)	
C15	0.10688 (16)	0.48525 (11)	0.24878 (9)	0.0322 (2)	
N1	−0.30636 (15)	0.20134 (11)	0.40532 (10)	0.0443 (3)	
N2	−0.22950 (16)	−0.10017 (13)	0.04030 (9)	0.0447 (3)	
N3	0.38541 (14)	0.53546 (10)	0.11487 (7)	0.0338 (2)	
N4	0.22586 (14)	0.55802 (10)	0.26462 (7)	0.0329 (2)	
N5	0.12224 (14)	0.42855 (10)	0.17511 (8)	0.0359 (2)	
N6	0.2906 (2)	0.40378 (15)	0.03568 (10)	0.0538 (3)	
N7	0.48129 (18)	0.65529 (12)	0.20504 (10)	0.0444 (3)	
N8	−0.03642 (18)	0.46571 (13)	0.31286 (10)	0.0465 (3)	
O1	−0.42179 (18)	−0.28988 (12)	0.38794 (9)	0.0562 (3)	
O2	−0.21683 (16)	0.19698 (11)	0.48256 (9)	0.0566 (3)	
O3	−0.37293 (19)	0.31089 (11)	0.33173 (12)	0.0782 (4)	
O4	0.05318 (15)	0.16839 (10)	0.26239 (8)	0.0464 (2)	
O5	−0.31476 (16)	−0.03215 (14)	−0.04647 (9)	0.0633 (3)	
O6	−0.19394 (18)	−0.22987 (12)	0.08023 (9)	0.0632 (3)	
O7	0.23235 (17)	0.53850 (11)	0.47450 (9)	0.0526 (3)	
H1	−0.357 (3)	−0.370 (3)	0.4389 (18)	0.093 (7)*	
H4	0.054 (3)	0.257 (2)	0.2186 (15)	0.071 (5)*	
H6A	0.216 (3)	0.353 (2)	0.0306 (14)	0.063 (5)*	
H6B	0.378 (3)	0.4227 (19)	−0.0065 (14)	0.060 (5)*	
H7A	0.243 (3)	0.555 (2)	0.4067 (17)	0.075 (6)*	
H7B	0.252 (3)	0.607 (2)	0.4863 (17)	0.084 (6)*	
H7C	0.576 (2)	0.6648 (17)	0.1643 (13)	0.054 (5)*	
H7D	0.478 (2)	0.6781 (18)	0.2619 (15)	0.061 (5)*	
H8A	−0.119 (2)	0.4277 (18)	0.3014 (14)	0.058 (5)*	
H8B	−0.048 (2)	0.4994 (19)	0.3622 (15)	0.062 (5)*	

### Atomic displacement parameters (Å^2^)

**Table:**

	*U*^11^	*U*^22^	*U*^33^	*U*^12^	*U*^13^	*U*^23^
C1	0.0295 (5)	0.0342 (6)	0.0400 (6)	−0.0099 (4)	0.0057 (4)	−0.0143 (5)
C2	0.0353 (5)	0.0380 (6)	0.0333 (6)	−0.0102 (5)	0.0008 (4)	−0.0151 (5)
C3	0.0442 (6)	0.0343 (6)	0.0329 (6)	−0.0094 (5)	0.0004 (5)	−0.0129 (5)
C4	0.0430 (6)	0.0430 (7)	0.0358 (6)	−0.0153 (5)	0.0064 (5)	−0.0214 (5)
C5	0.0385 (6)	0.0548 (8)	0.0365 (6)	−0.0139 (6)	−0.0014 (5)	−0.0188 (6)
C6	0.0329 (6)	0.0422 (7)	0.0389 (6)	−0.0057 (5)	−0.0015 (5)	−0.0083 (5)
C7	0.0374 (6)	0.0389 (6)	0.0356 (6)	−0.0163 (5)	0.0085 (5)	−0.0195 (5)
C8	0.0446 (6)	0.0391 (6)	0.0321 (6)	−0.0115 (5)	−0.0014 (5)	−0.0098 (5)
C9	0.0485 (7)	0.0279 (5)	0.0376 (6)	−0.0099 (5)	0.0000 (5)	−0.0093 (5)
C10	0.0371 (6)	0.0324 (5)	0.0344 (6)	−0.0131 (4)	0.0055 (4)	−0.0151 (5)
C11	0.0384 (6)	0.0312 (6)	0.0350 (6)	−0.0080 (5)	−0.0002 (5)	−0.0096 (5)
C12	0.0418 (6)	0.0271 (5)	0.0409 (6)	−0.0099 (4)	0.0070 (5)	−0.0135 (5)
C13	0.0448 (6)	0.0315 (5)	0.0297 (5)	−0.0136 (5)	−0.0005 (5)	−0.0134 (5)
C14	0.0394 (6)	0.0249 (5)	0.0281 (5)	−0.0092 (4)	−0.0024 (4)	−0.0100 (4)
C15	0.0380 (6)	0.0265 (5)	0.0317 (5)	−0.0073 (4)	0.0008 (4)	−0.0105 (4)
N1	0.0362 (5)	0.0353 (5)	0.0616 (7)	−0.0111 (4)	0.0050 (5)	−0.0169 (5)
N2	0.0466 (6)	0.0588 (7)	0.0475 (6)	−0.0282 (5)	0.0158 (5)	−0.0326 (6)
N3	0.0446 (5)	0.0344 (5)	0.0288 (5)	−0.0170 (4)	0.0032 (4)	−0.0148 (4)
N4	0.0412 (5)	0.0300 (5)	0.0322 (5)	−0.0104 (4)	0.0028 (4)	−0.0160 (4)
N5	0.0440 (5)	0.0357 (5)	0.0360 (5)	−0.0178 (4)	0.0036 (4)	−0.0175 (4)
N6	0.0714 (8)	0.0706 (8)	0.0509 (7)	−0.0428 (7)	0.0212 (6)	−0.0438 (7)
N7	0.0581 (7)	0.0516 (7)	0.0411 (6)	−0.0309 (6)	0.0084 (5)	−0.0269 (5)
N8	0.0490 (6)	0.0521 (7)	0.0498 (7)	−0.0222 (5)	0.0158 (5)	−0.0265 (6)
O1	0.0798 (7)	0.0498 (6)	0.0523 (6)	−0.0234 (6)	−0.0038 (6)	−0.0293 (5)
O2	0.0633 (6)	0.0503 (6)	0.0687 (7)	−0.0224 (5)	0.0021 (5)	−0.0310 (5)
O3	0.0767 (8)	0.0327 (5)	0.1046 (10)	−0.0105 (5)	−0.0261 (7)	−0.0024 (6)
O4	0.0633 (6)	0.0399 (5)	0.0426 (5)	−0.0224 (4)	−0.0035 (4)	−0.0166 (4)
O5	0.0642 (7)	0.0895 (9)	0.0527 (6)	−0.0272 (6)	−0.0010 (5)	−0.0398 (6)
O6	0.0876 (8)	0.0572 (6)	0.0702 (7)	−0.0425 (6)	0.0180 (6)	−0.0382 (6)
O7	0.0817 (7)	0.0497 (6)	0.0421 (6)	−0.0346 (5)	0.0143 (5)	−0.0242 (5)

### Geometric parameters (Å, º)

**Table:**

C1—C2	1.3766 (17)	C13—N6	1.3333 (16)
C1—C6	1.3877 (18)	C13—N3	1.3414 (14)
C1—N1	1.4501 (16)	C13—N5	1.3421 (16)
C2—C3	1.3774 (17)	C14—N7	1.3326 (15)
C2—H2	0.9300	C14—N3	1.3381 (14)
C3—C4	1.3857 (17)	C14—N4	1.3418 (16)
C3—H3	0.9300	C15—N8	1.3335 (17)
C4—O1	1.3538 (15)	C15—N4	1.3384 (15)
C4—C5	1.3852 (19)	C15—N5	1.3416 (15)
C5—C6	1.3726 (19)	N1—O3	1.2176 (16)
C5—H5	0.9300	N1—O2	1.2266 (16)
C6—H6	0.9300	N2—O5	1.2212 (17)
C7—C8	1.3804 (18)	N2—O6	1.2386 (17)
C7—C12	1.3815 (18)	N6—H6A	0.860 (18)
C7—N2	1.4518 (15)	N6—H6B	0.845 (18)
C8—C9	1.3770 (17)	N7—H7C	0.867 (17)
C8—H8	0.9300	N7—H7D	0.894 (19)
C9—C10	1.3871 (18)	N8—H8A	0.830 (17)
C9—H9	0.9300	N8—H8B	0.867 (19)
C10—O4	1.3488 (14)	O1—H1	0.90 (2)
C10—C11	1.3915 (17)	O4—H4	0.91 (2)
C11—C12	1.3747 (17)	O7—H7A	0.88 (2)
C11—H11	0.9300	O7—H7B	0.84 (2)
C12—H12	0.9300		
			
C2—C1—C6	122.08 (11)	C11—C12—C7	119.00 (11)
C2—C1—N1	118.88 (11)	C11—C12—H12	120.5
C6—C1—N1	119.04 (11)	C7—C12—H12	120.5
C1—C2—C3	118.59 (11)	N6—C13—N3	116.17 (11)
C1—C2—H2	120.7	N6—C13—N5	118.22 (11)
C3—C2—H2	120.7	N3—C13—N5	125.61 (10)
C2—C3—C4	120.13 (11)	N7—C14—N3	117.08 (11)
C2—C3—H3	119.9	N7—C14—N4	117.29 (10)
C4—C3—H3	119.9	N3—C14—N4	125.61 (10)
O1—C4—C5	117.44 (12)	N8—C15—N4	117.07 (11)
O1—C4—C3	122.07 (12)	N8—C15—N5	117.58 (11)
C5—C4—C3	120.48 (11)	N4—C15—N5	125.34 (10)
C6—C5—C4	119.93 (12)	O3—N1—O2	122.26 (12)
C6—C5—H5	120.0	O3—N1—C1	118.70 (12)
C4—C5—H5	120.0	O2—N1—C1	119.04 (11)
C5—C6—C1	118.78 (12)	O5—N2—O6	122.77 (11)
C5—C6—H6	120.6	O5—N2—C7	119.29 (12)
C1—C6—H6	120.6	O6—N2—C7	117.92 (12)
C8—C7—C12	121.83 (10)	C14—N3—C13	114.25 (10)
C8—C7—N2	119.31 (12)	C15—N4—C14	114.57 (10)
C12—C7—N2	118.84 (11)	C15—N5—C13	114.40 (9)
C9—C8—C7	118.99 (11)	C13—N6—H6A	118.8 (12)
C9—C8—H8	120.5	C13—N6—H6B	119.2 (12)
C7—C8—H8	120.5	H6A—N6—H6B	122.0 (17)
C8—C9—C10	119.96 (11)	C14—N7—H7C	118.7 (11)
C8—C9—H9	120.0	C14—N7—H7D	118.9 (11)
C10—C9—H9	120.0	H7C—N7—H7D	121.0 (15)
O4—C10—C9	122.67 (11)	C15—N8—H8A	119.2 (12)
O4—C10—C11	117.05 (11)	C15—N8—H8B	119.5 (11)
C9—C10—C11	120.27 (10)	H8A—N8—H8B	121.1 (16)
C12—C11—C10	119.93 (12)	C4—O1—H1	114.2 (14)
C12—C11—H11	120.0	C10—O4—H4	107.8 (12)
C10—C11—H11	120.0	H7A—O7—H7B	108.7 (19)
			
C6—C1—C2—C3	0.14 (18)	C2—C1—N1—O3	175.39 (13)
N1—C1—C2—C3	179.25 (11)	C6—C1—N1—O3	−5.47 (18)
C1—C2—C3—C4	−0.89 (18)	C2—C1—N1—O2	−3.93 (17)
C2—C3—C4—O1	−178.18 (11)	C6—C1—N1—O2	175.21 (11)
C2—C3—C4—C5	1.15 (18)	C8—C7—N2—O5	3.71 (17)
O1—C4—C5—C6	178.73 (12)	C12—C7—N2—O5	−174.89 (11)
C3—C4—C5—C6	−0.63 (19)	C8—C7—N2—O6	−177.86 (11)
C4—C5—C6—C1	−0.12 (18)	C12—C7—N2—O6	3.55 (17)
C2—C1—C6—C5	0.37 (18)	N7—C14—N3—C13	178.15 (11)
N1—C1—C6—C5	−178.74 (11)	N4—C14—N3—C13	−3.37 (16)
C12—C7—C8—C9	0.89 (18)	N6—C13—N3—C14	−176.36 (11)
N2—C7—C8—C9	−177.66 (11)	N5—C13—N3—C14	3.84 (17)
C7—C8—C9—C10	0.24 (19)	N8—C15—N4—C14	−177.10 (11)
C8—C9—C10—O4	179.76 (12)	N5—C15—N4—C14	4.13 (17)
C8—C9—C10—C11	−1.32 (18)	N7—C14—N4—C15	178.19 (11)
O4—C10—C11—C12	−179.73 (11)	N3—C14—N4—C15	−0.29 (16)
C9—C10—C11—C12	1.29 (18)	N8—C15—N5—C13	177.51 (11)
C10—C11—C12—C7	−0.18 (18)	N4—C15—N5—C13	−3.73 (17)
C8—C7—C12—C11	−0.92 (18)	N6—C13—N5—C15	179.61 (12)
N2—C7—C12—C11	177.64 (10)	N3—C13—N5—C15	−0.59 (17)

### Hydrogen-bond geometry (Å, º)

**Table:**

*D*—H···*A*	*D*—H	H···*A*	*D*···*A*	*D*—H···*A*
O1—H1···O7^i^	0.90 (2)	1.76 (2)	2.6600 (18)	172 (2)
O4—H4···N5	0.91 (2)	1.87 (2)	2.7217 (16)	157 (2)
N6—H6*A*···O6^ii^	0.860 (18)	2.363 (19)	3.0276 (16)	134 (2)
N6—H6*B*···N3^iii^	0.845 (18)	2.235 (19)	3.080 (2)	178 (2)
O7—H7*A*···N4	0.88 (2)	1.94 (2)	2.8020 (18)	166 (2)
O7—H7*B*···O2^iv^	0.84 (2)	2.22 (2)	3.0424 (18)	164 (2)
N7—H7*C*···O6^v^	0.867 (17)	2.250 (17)	3.056 (2)	155 (2)
N7—H7*D*···O1^v^	0.894 (19)	2.049 (19)	2.8996 (17)	159 (2)
N8—H8*A*···O3	0.830 (17)	2.367 (18)	3.158 (2)	159 (2)
N8—H8*B*···O7^iv^	0.867 (19)	2.517 (18)	3.1890 (19)	135 (2)

Symmetry codes: (i) −*x*, −*y*, −*z*+1; (ii) −*x*, −*y*, −*z*; (iii) −*x*+1, −*y*+1, −*z*; (iv) −*x*, −*y*+1, −*z*+1; (v) *x*+1, *y*+1, *z*.
